# Validation of response processes in medical assessment using an explanatory item response model

**DOI:** 10.1186/s12909-022-03942-2

**Published:** 2022-12-10

**Authors:** Veerapong Vattanavanit, Sungworn Ngudgratoke, Purimpratch Khaninphasut

**Affiliations:** 1 Educational Measurement and Evaluation Program, School of Educational Studies, Sukhothai Thammathirat Open University, Nonthaburi, 11120 Thailand; 2 Faculty of Medicine, Prince of Songkla University, 15 Kanjanavanich Road, Hat Yai, Songkhla, 90110 Thailand

**Keywords:** Validity, Response process, Cognitive load, Difficulty

## Abstract

**Background:**

Response process validation is a crucial source of test validity. The expected cognitive load scale was created based on the reflection of the mental effort by which borderline students solve an item defined by experts. The stem length affects the students’ extraneous cognitive load. The purposes of this study were to develop an exam for medical students and corroborate the response process validity by analyzing the correlation between the expected cognitive load, stem length, and the difficulty.

**Methods:**

This was a correlational study. Five medical teachers as the experts and 183 third-year medical students were enrolled from the Faculty of Medicine, Prince of Songkla University, Thailand. The instruments used were a medical physiology exam and a three-level expected cognitive load evaluation form judged by medical teachers. Data were analyzed using an explanatory item response model.

**Results:**

The test consists of 20 items and 21 possible scores. The median score was 8, with a quartile deviation of 1.5. Nine items had long stems (more than two lines). Sixteen items were judged as high (level 2 or 3) expected cognitive load. When adding the expected cognitive load in a Rasch model, the expected cognitive load significantly correlated with item difficulty. In the Rasch model that included both the expected cognitive load and stem length, a long stem had a greater effect on item difficulty than low expected cognitive load. However, the Rasch model showed the best fit.

**Conclusions:**

The long stem had a stronger correlation with test difficulty than expected cognitive load, which indirectly implied response process validity. We suggest incorporating stem length and expected cognitive load to create an appropriate distribution of the difficulty of the entire test.

**Supplementary Information:**

The online version contains supplementary material available at 10.1186/s12909-022-03942-2.

## Background

The key question when testing is how well the scores are used to represent what is being measured. The degree of evidence that supports the interpretation of test scores for proposed uses of tests is called “validity” [1]. The three subtypes of validity in the conventional perspective are content, criterion-related, and construct. Validity has recently been reconceptualized as a unitary aspect known as “construct validity.” Five sources of construct validity evidence have been specified: test content; response processes; internal structure; relations to other variables; and consequences of testing [1]. One of the sources of validity evidence is the response process, which is the study of whether the examinees’ responses were obtained through appropriate processes of thinking or consistent with the assumptions of examiners. The cognitive response processes for the exam include how information is accessed, represented, revised, acquired, and stored to answer a question [2]. There are various response process investigations, such as think-aloud, response times, interviews, and eye-tracking methods [3]. Different methods of assessments directly affect response process validity. Computer-based assessment is an electronic assessment tool that has reduced the burden on teachers and facilities to conduct examinations purposefully. These advances have been in the context of computer-based assessments of explanations and think-aloud protocols during reading comprehension. These automated systems incorporate a variety of natural language processing tools and algorithms to assess the responses [4]. A study found that the expected gender effect on reading ability coincides with a gender effect in speed in computer-based assessments but not in paper-based assessments [5]. However, evidence of the validity of these response processes must be obtained during the exams or after the examinees have completed the tests.

When the examinees start taking the test, they use the thinking process and extract part of the memory from their brains. The limit of a person’s working memory to perform a particular skill is known as the “cognitive load” [6]. Cognitive load theory and applications have been studied extensively to facilitate medical education. The theory suggests that there are two main types of cognitive load: intrinsic and extraneous load [7]. Intrinsic load is based on the nature of learning material that relates to the difficulty of the item itself. Extraneous load focuses on anything in irrelevant instructional materials that occupies working memory capacity. There are cognitive load subjective assessment tools, such as the Paas scale [8], in which examinees assess themselves on how much mental effort it takes to solve a problem. The Paas scale is a nine-point Likert scale, with level 1 defined as requiring very little mental effort and level 9 defined as requiring very high mental effort. The self-report cognitive load was related to the difficulty of the exams [9]. Reducing the examinees' cognitive load by adjusting the exams to a more understandable format, shortening the stem by cutting out unnecessary content, and having illustrations reduce the extraneous cognitive load, which results in examinees doing better in the exam [10].

The authors adapted the self-report cognitive load measurement tool to an expected cognitive load measurement, which measures the cognitive load required by borderline or minimally acceptable examinees to complete the exam judged by experts. The mock exam is based on the summative exam to study the relationship between the expected cognitive load and the test difficulty. We hypothesized that the probabilities of answering the exam correctly among medical students depended on two main properties: the item and the students. The item properties consist of the difficulty according to the Rasch model, the expected cognitive load for each item, and the length of stems that reflect the extraneous cognitive load. The students’ properties involve their ability. The test difficulty can be improved by additional variables using an explanatory item response model (EIRM). The expected cognitive load assessed by experts, and stem lengths that are well correlated with test difficulty and the likelihood of correct answers, will be indirect evidence of the response process validity.

## Methods

### Participants

Participants were experts and medical students from the Faculty of Medicine, Prince of Songkla University, Thailand. Five experts were purposively selected from medical teachers with more than five years of experience in cardiovascular or pulmonary physiology teaching. All third-year medical students were invited to take the test. Since participation in the study was voluntary, only medical teachers and students who gave consent for voluntary participation were included in the study.

### Test and evaluation form development

The test for medical students was created using five-option multiple-choice questions. Test contents were based on respiratory and cardiovascular physiology, which in the part of the summative exam followed the Medical Council of Thailand and the World Federation for Medical Education (WFME) standard [11]. The test was written in English. All test items passed the content validity testing by qualified experts. After a pilot study with 40 medical students, 20 items with an appropriate difficulty index (*p* = 0.2–0.8) and discrimination index (r > 0.2) were selected [12]. The Cronbach’s alpha reliability of the test was 0.833. Regarding the length of stems as one of the item properties, a long-stem item is defined as an item of more than two lines or 212 characters with spaces; there were nine such items included in our test. For convenience, stem length was collapsed into two categories – long and short stems – as easy-to-remember tools for designing appropriate tests.

The expected cognitive load scale is adapted from the subjective ratings of total cognitive load [8]. The expected cognitive load is defined as the mental effort that borderline students (a group that has a 50% chance of passing [13]) use to complete the item, which ranges from levels 1 to 3. Level 1 is defined as low mental effort; Level 2 is defined as neither low nor high mental effort; and Level 3 is defined as high mental effort. The expected cognitive load evaluation forms including items, corrected answers, and the expected cognitive load scale for each item were provided to the experts. (Additional file 1).

### Data collection

The authors invited five medical teachers as the experts to complete the expected cognitive load evaluation forms. We provided exam details to all third-year medical students and invited voluntary participants to join an online exam via Zoom on February 22, 2022. With proctoring by the authors and education officers, students were allowed to complete the exam in 30 min. Data of item and student properties, including the length of items, the expected cognitive load for each item, the identified number of students, as well as their answers for each question, were collected in the long data format.

### Statistical analysis

Our item analysis relied on classical test theory and item response analysis. To estimate the influence of the expected cognitive load and the length of stems on an item’s difficulty, the EIRM was applied. The EIRM belongs to the Rasch models (one-parameter item response models), a family of established psychometric models utilized in education research. The EIRM aims to explain how item properties (the expected cognitive load, length of items) and personal characteristic (students’ ability) affect the students’ responses to items. An EIRM that explains item properties is called an item explanatory model or linear logistic test model (LLTM) [14].

In this study, we analyzed three models. Model 1 was based on the Rasch model without adding any item variables. The result from Model 1 was item difficulty. The other two models were LLTMs with the specified variables to estimate difficulty parameters. Model 2 included test property and the expected cognitive load in Model 1. Model 3 combined the expected cognitive load and the length of stems into Model 1. The Akaike information criteria (AIC), Bayes information criteria (BIC), and log-likelihood difference test were applied to compare the fit of all models [15].

The minimum sample size of 150 students in the setting of a 20-item test length is sufficient to estimate item parameters accurately in the dichotomous item response model [16].

The correlation between expected cognitive load or stem length and proportion-correct scores was calculated using a Spearman correlation coefficient and Eta coefficient. A Phi coefficient for the correlation between expected cognitive load and stem length was calculated.

The statistical analysis was performed with the use of SPSS software (SPSS for Windows, version 28.0) and R software, version 4.2.1 (R Foundation for Statistical Computing), using the eirm package for EIRMs [17]. (Additional file 2).

## Results

### Exam results

Of 191 medical students, 183 completed the test (95.81%). Most of the students were female (121/183, 66.1%).

The highest score was 20, and the lowest score was 3. The median score was 8, with a quartile deviation of 1.5. The distribution of test scores was slightly skewed to the right (skewness 0.85, kurtosis 1.26).

The exam difficulty index (p) ranges from 0.17 to 0.69. The most difficult item was item 9, and the easiest item was item 4. The discrimination index (r) ranged from 0.08 to 0.57. Three items (2, 9, and 16) had an r less than 0.2.

Four items (4, 6, 8, and 11) had the expected cognitive load level of 1. Only two items (10 and 20) had the expected cognitive level of 3, and the other 14 items had the expected cognitive level of 2. Therefore, we decided to combine the Level 2 and 3 expected cognitive load items as “high” expected cognitive load items and Level 1 as “low” expected cognitive load items. Expected cognitive load was collapsed into two categories for two reasons. First, there were a small number of items (2 items) of Level 3 cognitive load. Second, for analytical issues, the test had only 20 items, which did not fully represent every scale of expected cognitive load judged by the experts.

### Data analysis using an explanatory response model

**Fig. 1:**
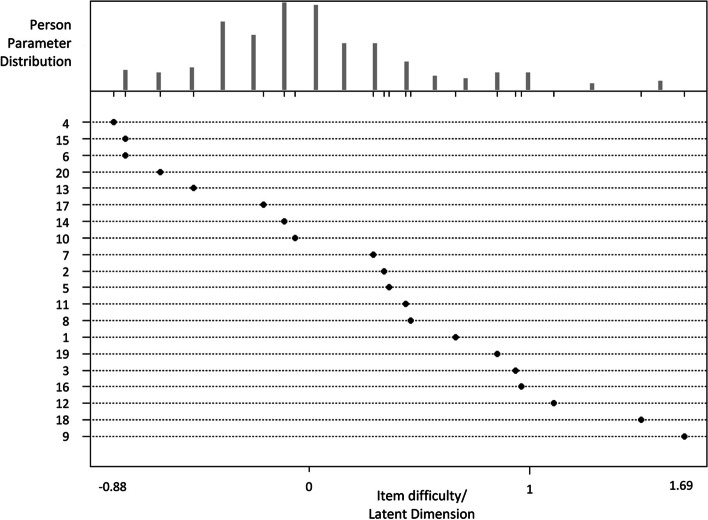
Person-item map of Model 1. Note: The person-item map displays the location of personal abilities and item difficulties along the same latent dimension. The person parameter is located on the scale from left (low ability) to right (high ability). Locations of item difficulties are displayed with solid circles from the easiest on the left to the most difficult on the right

Model 1 was an analysis of the exams according to the Rasch model. In an item response theory analysis, trait levels and item difficulties are usually scored on a standardized metric with a mean of 0 and a standard deviation of 1. A student who has a trait level of 0 has an average level of that trait. Similarly, an item with a difficulty level of 0 is an average item. The results revealed that the test difficulty (b) was in the range of -0.88 to 1.69, with the most difficult item being item 9 and the easiest being item 4. The distribution of students’ abilities as well as the difficulty are shown in the person-item map in Fig. 1.

Model 2 added the expected cognitive load into Model 1. The estimated fixed effect of two levels of the expected cognitive load on the difficulty is shown in Table 1. The items with low expected cognitive loads had a difficulty level significantly lower than zero. On the contrary, items with high expected cognitive loads had a difficulty level significantly higher than zero.

**Table 1:** Fixed effect of the expected cognitive load on the difficulty

Variables	Difficulty	SE	*P* value
Low expected cognitive load	-0.18	0.08	0.029
High expected cognitive load	0.35	0.05	< 0.001

Abbreviation: *SE* standard error

**Fig. 2:**
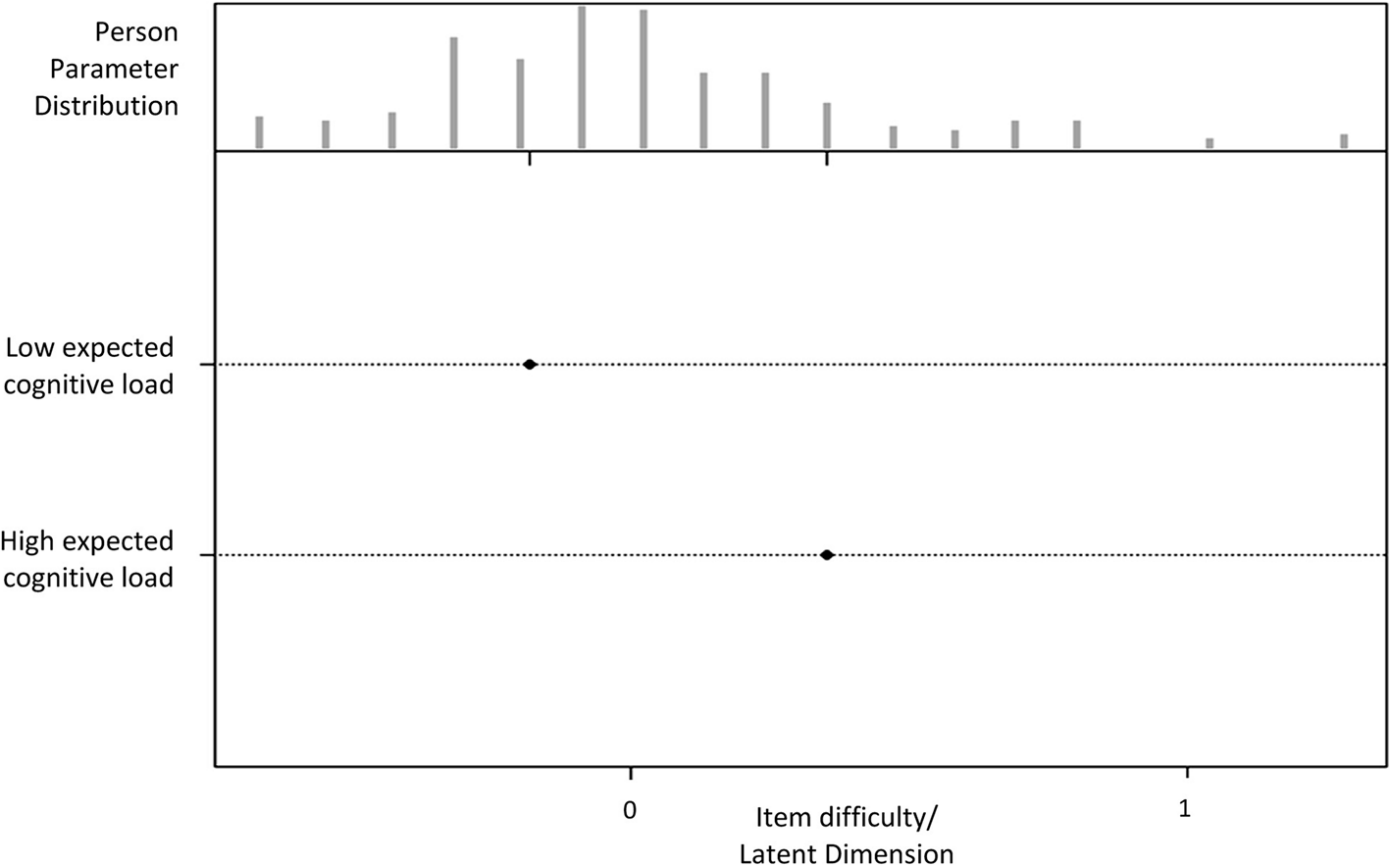
Person-item map of Model 2

The results from Model 2 can be displayed as a person-item map, as shown in Fig. 2.

Model 3 added both test properties – the expected cognitive load, and length of stems – into Model 1. The estimated fixed effect of both test properties on the difficulty is shown in Table 2. Significantly, the items with low expected cognitive loads had a difficulty level lower than zero, while items with long stems had a difficulty level higher than zero. Compared to the low expected cognitive load, the long stem had a greater effect on item difficulty.

**Table 2:** Fixed effect of the expected cognitive load and long stem on the difficulty

Variables	Difficulty	SE	*P* value
Low expected cognitive load	-0.38	< 0.001	< 0.001
High expected cognitive load	-0.02	0.696	0.69
Long stem	0.78	< 0.001	< 0.001

Abbreviation: *SE* standard error

**Fig. 3:**
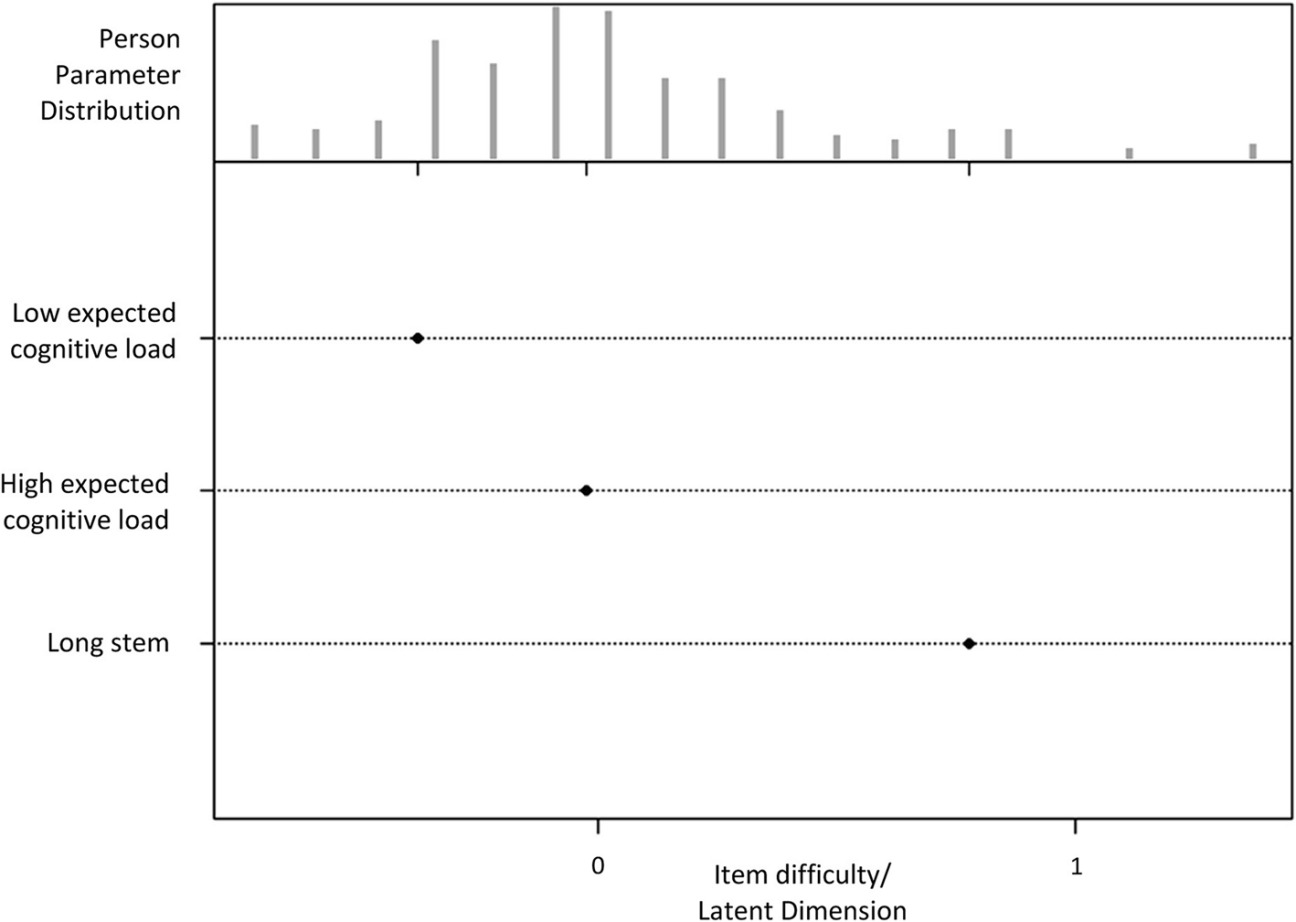
Person-item map of Model 3

The results from Model 3 can be presented as a person-item map, as shown in Fig. 3.

The AIC, BIC, and log-likelihood difference test for model comparison between the Rasch (one-parameter model) and the two LLTMs are shown in Table 3. The smaller values of the AIC and BIC indicate a better fitting model. These values therefore indicate that Model 1 fits better with the data than Model 2 or Model 3. The log-likelihood difference test also proposes a significantly better fit of the Rasch model compared to both LLTMs. Comparing both LLTMs, AIC and BIC indicate that the LLTM with combined expected cognitive load and long stem fits better to the data than the LLTM with only the expected cognitive load.

**Table 3:** Comparison of the three models

Model	AIC	BIC	Log-likelihood	Deviance	Random effect (intercept)	
					Variance	SD
1 (Rasch)	4580.9	4711.2	-2269.5	4538.9	0.3379	0.5813
2 (expected cognitive load)	4931.0	4949.6	-2462.5	4925.0	0.2429	0.4928
3 (expected cognitive load + long stem)	4815.0	4839.8	-2403.5	4807.0	0.2689	0.5186

Abbreviations: *AIC* Akaike information criteria, *BIC* Bayesian information criteria, *SD* standard deviation

### Correlation of expected cognitive load, stem length, and proportion-correct scores

The correlation of expected cognitive load and stem length with proportion-correct scores in the classical item analysis are shown in Table 4.

**Table 4:** Correlation of expected cognitive load, stem length, and proportion-correct scores

	**Proportion-correct scores**	**Expected cognitive load**	**Stem length**
Proportion-correct scores	1.00		
Expected cognitive load	-2.93^a^	1.00	
Stem length	.58^b^	.20^c^	1.00

^a^Spearman correlation

^b^Eta coefficient

^c^Phi coefficient

Expected cognitive load showed an insignificant negative correlation with proportion-correct scores (*ρ* = -2.93, *P* = 0.210). Stem length revealed an insignificant positive correlation with proportion-correct scores (η = 0.58, *P* = 0.390). Stem length and expected cognitive load had an insignificant positive correlation (ϕ = 0.20, *P* = 0.369).

## Discussion

Our test was created as a summative test for third-year medical students. The test difficulty (b) was in the range of -0.88 to 1.69, indicating an average level (-2.0 to 2.0) of difficulty [12]. Our students’ median score did not reach 50%. The respiratory and cardiovascular physiology content selected for this exam is one of the difficult parts of medical physiology. Taking a physiology exam requires students’ memory, reasoning, calculation, and integration of data [18]. Moreover, the exam was written in English for non-native speaker examinees. Regarding the language barrier, a study by Phisalprapa et al. [19] examined the results of 120 multiple-choice questions of fourth-year medical students at the Faculty of Medicine Siriraj Hospital. The results found that the medical students answered the exam correctly when the exam was written in Thai compared to the English exam, especially among borderline students. According to the policy of our medical school and the comprehensive exam-constructing guidelines of the Center of Medical Competency Assessment and Accreditation, the summative multiple-choice questions (MCQs) for medical students should be in English. The impact of language on test scores in both clinical practice testing and paper-based testing, particularly regarding MCQs, has been the subject of numerous reports from all over the world [20, 21]. Thai medical students continue to experience issues with English language-related medical skills testing in medical school, including longer test-taking times and poorer scores than material assessed in the Thai language, while having excellent English test scores compared to other student groups [19]. A study revealed that foreign language exams increased cognitive load and learning academic content in university students [22]. However, we realized that our test aim was to test medical knowledge, not language skills. So, we tried to use simple words in every item as much as possible.

The expected cognitive load scores are statistically related to item difficulty in Model 2. This is consistent with the authors’ hypothesis. Previous studies mentioned the cognitive load assessed directly by the examiners – such as the time spent on the test [23], or the examinees’ self-report [24] – was correlated with test difficulty. However, previous studies used the cognitive load scale with different Likert scales, as determined by the investigator. To our knowledge, this is the first study using the expected cognitive load judged by experts to assess or predict how much mental effort examinees will spend on each test item. In addition to the expected cognitive load, long-stem items were related to the difficulty of the test. To complete the long-stem items, a lot of attention was required from examinees. Kettler et al. [25] found that reducing the length of the items reduced the difficulty of the exams. Gillmor et al. [10] found that trying to reduce the cognitive load of the exam by reducing the length of items and making the test easier to read resulted in correct answers.

When the long-stem variable was added to the LLTM, only the low expected cognitive load and long stem were statistically correlated with the difficulty. The long stem has a stronger correlation with difficulty than expected cognitive load because it is a constant that does not depend on judgment. And when testing the model fit, it was found that the Rasch model was the fittest, followed by a model that included the expected cognitive load and a long stem. The reason for this may be that long stems increase the cognitive load on examinees and affect the difficulty. Therefore, a long stem was directly associated with a high level of expected cognitive load. Bringing together variables into the model, the high expected cognitive load was reduced in the fixed effect on the difficulty. Nevertheless, we could not find a significant correlation of expected cognitive load or stem length with proportion-correct scores in the classical item analysis. The possible reason for this might be due to the very small number of items which gives a low power to detect correlation. Moreover, there may also be other random variables related to difficulty in addition to our interesting properties. Further analysis of the random item effect with LLTM may make the model more consistent, according to Hartig et al. [26].

There were some limitations encountered in using the expected cognitive load ​​in this study. First, the expected cognitive load of items judged by experts was mostly graded on a scale of 2, with a very low rating on a scale of 3. This drove the authors to combine the expected cognitive load of levels 2 and 3 into only two scales: low and high expected cognitive load. The values ​​obtained may affect the validity of the assessment. Preston and Colman [27] studied the appropriate rating scales to determine what level they should be. In a case in which 149 respondents assessed the satisfaction with restaurants they had recently visited on a scale ranging from 2 to 11 and 101 levels, the test–retest reliability was lowest for two-point, three-point, and four-point scales. Internal consistency was lowest for two or three scales and highest for those with seven or more.

Second, the reliability of the experts should be considered. Experts independently assessed the expected cognitive load and scaled each item. The authors analyzed inter-rater reliability by testing Cohen’s kappa coefficient. The mean was 0.515, meaning that the reliability between the evaluators was weak [28].

Third, the validity of the response process that the authors studied was not directly measured by the medical students. This process of the expected cognitive load estimation was similar to the Angoff method [29] in cut score evaluation, in which experts imagine how many out of 100 borderline examinees will be able to answer each question correctly. There is a collective opinion of experts, which may result in more reliability than the assessment in our study. However, some researchers are skeptical of the subjective assessment of the cognitive load and criticize that estimating this mental effort is an impossible mission [30].

Our results revealed that a long stem has a greater effect on difficulty than expected cognitive load. Collecting expected cognitive load necessitates expert information, which takes time and resources. So, we suggest incorporating stem length stems to create an appropriate distribution of the difficulty of the entire test in setting a time or resource limit.

### Future recommendation

The authors suggest increasing the validity of the expected cognitive load assessment through experts’ meetings to define an appropriate scale for borderline students. An additional random effect could be analyzed in the LLTM for improving the model fit. It may also be worthwhile to compare the indirect validity with the response process measured directly, such as self-report cognitive load, response time, or think-aloud interview.

## Conclusions

The long stem had a stronger correlation with test difficulty than expected cognitive load, which inferred the validity of the response processes. We recommend using stem length and/or expected cognitive load in the selection of appropriate test items.

## Supplementary Information

**Additional file 1.** Example of the expected cognitive load evaluation form

**Additional file 2.** R syntax for an explanatory response model

## Data Availability

The data from this study are available from the corresponding author upon request.

## Abbreviations

AIC: Akaike information criteria
b: Test difficulty
BIC: Bayesian information criteria
EIRM: Explanatory item response model
LLTM: Linear logistic test model
MCQs: Multiple-choice questions
p: Difficulty index
r: Discrimination index
SD: Standard deviation
SE: Standard error
