# Supplementary material for: Validation of response processes in medical assessment using an explanatory item response model
Source: BMC Med Educ. 2022 Dec 10;22:855. doi: 10.1186/s12909-022-03942-2 (PMC9737731; doi:10.1186/s12909-022-03942-2)
Supplement: Supplementary file 1 — Additional file 1. Example of the expected cognitive load evaluation form [file 12909_2022_3942_MOESM1_ESM.docx]

**Additional file 1**

**Example of the expected cognitive load evaluation form**

| Item | 3 | |
| --- | --- | --- |
| **CODE/Topic: B 6.1.2.2 ventilation** | | |
| **Stem & Options**  A 65-year-old man with a history of COPD is admitted to the hospital due to acute respiratory failure from COVID-19 infection. An analysis of arterial blood gases shows that his PO_2_ is 60 mm Hg and his PCO_2_ is 70 mm Hg. His exhaled minute ventilation rate is two times higher than that of a normal individual of the same age and body size. Why does he have hypercapnia despite having an increased exhaled minute ventilation rate?   1. Alveolar ventilation is increased 2. Dead space ventilation is increased 3. Tidal volume is increased 4. Ventilation/perfusion ratio is decreased 5. Intrapulmonary shunt is increased | | |
| **Answer** | **B** | The patient has a decreased alveolar ventilation rate, evidenced by the increased arterial PCO_2_. Decreased alveolar ventilation in the presence of increased total exhaled minute ventilation can only be explained if dead space ventilation is increased. |
| **Predicted cognitive load** | **2** |  |
| **Taxonomy** | 🗹 Application of knowledge 🞏 Recall | |

**Expert opinion**

Choose the expected cognitive load in your opinion

🞏 1 🞏 2 🞏 3

**Note**

1 = low mental effort

2 = neither low nor high mental effort

3 = high mental effort
