# Supplementary material for: Validation of response processes in medical assessment using an explanatory item response model
Source: BMC Med Educ. 2022 Dec 10;22:855. doi: 10.1186/s12909-022-03942-2 (PMC9737731; doi:10.1186/s12909-022-03942-2)
Supplement: Supplementary file 2 — Additional file 2. R syntax for an explanatory response model [file 12909_2022_3942_MOESM2_ESM.docx]

**Additional file 2**

**R syntax for an explanatory response model**

# Install and activate the package

install.packages("eirm")

library("eirm")

#-------------- Dichotomous EIRM ----------------------------#

# Rasch model (baseline model)

rasch <- eirm(formula = "response ~ -1 + item + (1|student)", data = dat1)

print(rasch, difficulty = TRUE) # Difficulty

plot(rasch, difficulty = TRUE) # Person-item map with difficulty

# Linear Logistic Test Model (LLTM)

lltm <- eirm(formula = "response ~ -1 + cognitive + word + (1|student)", data = dat1)

print(lltm, difficulty = TRUE) # Difficulty

plot(lltm, difficulty = TRUE) # Person-item map with difficulty

# Model fit indices

summary(rasch$model)

summary(lltm$model)
